# A Gynæcological Case

**Published:** 1888-08

**Authors:** E. W. Berridge

**Affiliations:** London


### A GYNÆCOLOGICAL CASE.

E. W. BERRIDGE, M. D., LONDON.

January 16th, 1883, a lady recommended to me by Dr. Bayard, of New York, wrote to me respecting her daughter, who had been married two months, and was probably pregnant. The symptoms given were as follows: Dull, constant pain, indicating prolapsus uteri; also for the last week headache, dizziness, loss of appetite, and chilliness: though naturally playful, now seems very desponding. I prescribed *Pulsatilla*$^{cm}$ (F. C.), a dose every morning for seven days.

January 23d, the mother wrote that the uterine pains were much relieved, but the nausea (not mentioned in her former letter) grew worse; little appetite, headache over eyes, coated tongue, skin looking bilious, much depressed in spirits; nothing remained long on stomach, and the nausea sometimes continued at night. As the indications were not clear, I prescribed no more medicine till she could see me.

February 4th, I saw her in London and took the following *photo:* Nausea soon after waking. If she can vomit white froth before any meal she can retain the meal, but not otherwise. Nausea comes on at any time. Yesterday had longing for peas or beans; for a week or two has had at times craving for vegetables. Is more cheerful since seeing her friends in London. Thirsty for little at a time, but cold water causes nausea. Likes warm drinks better than cold. About five or six years ago had backaches, and Drs. Swan and Bayard diagnosed kidney mischief. Always has had frequent desire to urinate, and now it is much worse, coming on continually, sometimes without result. Dull pain in lower abdomen before urinating (used to have this symptom even when in ordinary health, if she had to refrain from urinating); after urinating, shooting in left abdomen from behind forward, on the same level as the dull pain, lasting from three to four minutes, and followed by the dull pain again. If she lies on left side feels a sore spot in left abdomen; better by lying on right side, and especially better if something is placed between knees. Last menses commenced on December 13th, but much less than usual. Hands and feet cold. Constipated for over two weeks; has to use a cold water enema daily. Dreams much about robbers and wild animals, very vivid, and the impression lasts after waking.

Any movement makes her feel chilly between scapulæ with creeping there, followed by chilliness in stomach, going backward into her. Sore throat if she gets her feet wet.

*Diagnosis of remedy.*—I took the peculiar dreams as the key-note of the case, on account of their close relationship to mental symptoms.

Dreams about robbers belong to *Alum.*, *Aur.*, *Bell.*, *Calc-ph.*, *Castoreum*, *Ferr-iod.*, *Jacaranda*, *Kali-c.*, *Lilium-c.*, *Magn-c.*, *Magn-m.*, *Magn-s.*, *Merc-sol.*, *Merc-iod-rub.*, *Merc-sol.*, *Natr-c.*, *Natr-m.*, *Petr.*, *Phell.*, *Phosph.*, *Plumb.*, *Psor.*, *Ptel.*, *Rumex*, *Sanicula*, *Silic.*, *Verat.*, *Zinc.*

*Dreams of wild animals, *Daphn-ind.*, *Hydrast.*, *Hyos.*, *Indium*, *Lycop.*, *Merc.*, *Phos.*, Puls., *Sulph.*, *Tarent.*

---

* NOTE.—In Allen's *Index*, p. 813. Dreams of crocodiles is attributed to *Ledum palustre;* it should be *Sedinha.*

Dreams continuing after waking, *Calc.*, *China*, *Ign.*, *Natr-c.*, *Natr-m.*

This reduces the number to Natr-c., *Natr-m.*, *Phos.*, *Puls.* Pain in abdomen after urinating belongs to *Chelid.*, *China*, *Magn-c.*, *Natr-mur.*, *Staph.*, *Sulph-ac.*

This reduces the number to Natr-mur., which also has chilliness when moving, and nausea from drinking. The other symptoms I could not find under this remedy, but selected it as corresponding to the greatest number and the most important. To leave off salt (℞ *Natr-mur.*$^{1m}$, Jenichen) twice daily for six days.

February 6th.—Sends the following report by letter: Urinary symptoms have materially abated, but the pain in left side seems worse; this latter pain is in region of left ovary, as if something had become attached to the walls of abdomen, and the extension of any muscle there gives a pain as if stitches were being ripped. The constant pain is a pulling, ripping pain, and yesterday it was so much worse that the whole left leg as far as the knee felt numb. A year ago had the same pain in a less degree, and was examined by Dr. Webster, a lady physician, who discovered slight enlargement of left ovary. Yesterday a new pain came on; when she has been for sometime allowing her left side to sustain its equal share of the weight of the body, a pain comes on parallel with the vagina, but quite to the left; it is sharp, but not shooting; it does not move, but seems to come at once into its entire course, as if it were wedge-shaped, the point being in region of left ovary, and the base quite to the left of the orifice of vagina; this pain was very frequent yesterday. Also yesterday, when walking, constant feeling as if she was going to fall; no giddiness, but it seemed as if at any movement her heels would rise off the ground and that she would fall stiffly on her forehead. Yesterday it was only once, before breakfast, necessary to relieve the stomach of the froth. Languid yesterday, and did not sleep well last night. This morning vomited pure bile, instead of the white froth, before breakfast. Yesterday severe headache; it seemed to be in the very bones, increased by cold air, and ameliorated by any warm applications to forehead; to-day it has quite gone; she never had such headaches in the United States. Continue medicine.

February 13th.—Her mother writes: Has been much better till this morning. Has had no medicine since February 10th. Has not been sick till to-day, except morning and evening, but now has nausea almost always, and feels very languid in mind and body. The pains are better, but the wedge-shaped pain comes on at times, especially at night, so severe as to affect the left leg. Headache, with general feeling of enlarging and pressure on vertex. Light dim after using eyes a short time. Constant desire to have occiput supported.

February 17th visited me again—no medicine—and gave the following report: Sickness much better, only before breakfast, and one day it did not take place at all. Feels better. The pulling, ripping, adhesive-feeling pains are quite gone. The wedge-pain is only a little better; it is worse at night, especially when lying on left side, and it causes cramp in back of left thigh, especially at night and worse then if lying on left side. Still some headache. Constant desire to have occiput supported, which relieves pressure in occiput and down back. Feeling of falling is gone. More constipation. Less dreaming; no dreams of robbers or animals, and the impression no longer lasts after waking. Chilliness gone. Feet are still cold. No unnatural thirst. Urination quite normal. Sore pain in abdomen gone. Eats no raw salt now. Bruised pain in left natis, burning and sharp pain going about an inch upward and inward when sitting on that side.

As the former remedy appeared to have ceased to act, and a new symptom of importance arisen, I took it as the key-note for the next prescription. *Causticum* is the only remedy I could find having bruised pain in nates when sitting; and though I could not find under it either the wedge-pain or the desire to support occiput, yet as it corresponded to the latest symptom it was evidently the *simillimum*.

Causticum$^{cm}$ (Fincke) every morning for seven days. March 6th wrote to say she was feeling quite well, with great improvement in all her symptoms, till she took a bad cold, which now confined her to bed for two days. Dry, hollow cough, sounding like croup; pain in left lung when coughing; throat very sore, most inflamed on left side. Skin hot and dry. Eats well, and retains her food. Last night great dyspnœa and choking in throat. Has taken Aconite,* but with only slight relief.

Lachesis$^{cm}$ (F. C.) every three hours for four days. March 14th, the *Lachesis* did some good, but the catarrh has brought back some of the former symptoms. Bruised pain in left natis returned, but not so sharp as formerly, on sitting; worse by coughing, especially if she is standing. Bowels move regularly,

* "Taking cold the cause of half our diseases." Specific, *Aconite*. But, unfortunately for the reputation of the discoverer, it does not answer unless the symptoms correspond.

but stools too large and painful. No urinary trouble. Every morning vomits froth, but not food, and very seldom does nausea accompany it. Not much dreaming now. Chilliness gone. Pain in left ovary had ceased, but has returned a little; no wedge-pain now. No feeling of falling. Cough caused by feeling of a ball in throat, with sputa like white of egg; cough worse in cold air, but *better* by eating and by talking. Feels mentally and physically fatigued. Hiccough troublesome at times. *Causticum*$^{cm}$ (Swan) every four hours for a week.

April 14th writes that all the old pains have gone, though once or twice after over-fatigue they returned with some severity, passing off in a day or two. Sickness every morning with occasional exceptions, but no nausea; it is still white froth, seldom any bile, and there are now four or five greenish spots in it. Often troubled after eating, and also when she has been sometimes without a meal, with a burning extending from roof of mouth to stomach. At times burning sour taste in mouth. Constipation; stools very painful. After rising in morning and moving about, sensation of an ice-cold wind blowing against her in region of stomach, and extending over the breast; the pain at times is excruciating, and reaches its climax at the nipple. During the past week this pain has been somewhat relieved by wearing a piece of flannel. These sensations occur at times *from change of motion*, but are never so exquisitely painful as in the morning, and they never occur at night. Irritability, which she controls with effort, and a longing to tear or break something; this last symptom she has often had before, but it is now peculiarly strong; the motive is not spiteful. Her younger brother has this symptom, and says it relieves him to break things.

Diagnosis of remedy:

Destroys things.—*Agar.*, *Ananth.*, *Bell.*, *Bufo*, *Canth.*, *Carb-sulph.*, *Cubebs*, *Hyos.*, *Laches.*, *Laur.*, *Merc-iod-flav.*, *Moschus*, *Phosph.*, *Plumb.*, *Stram.*, *Solan-tub-œgr.*, *Stront.*, *Sulph.*, *Tarent.*, *Verat.*

Breaks things.—*Hura*, *Solan-tub-œgr.*

Tears things.—*Bell.*, *Camph.*, *Hyos.*, *Op.*, *Phosph.*, *Stram.*, *Sulph.*, *Verat.*

Frothy vomit.—*Acon.*, *Æth.*, *Ars.*, *Arund-m.*, *Canth.*, *Cepa*, *Cic-m.*, *Croton*, *Cupr.*, Ferr., *Kali-b.*, *Kali-oxal.*, *Kali-sulfuratum*, *Lycop.*, *Merc-corr.*, *Morph.*, *Mur-ac.*, *Natr-c.*, *Op.*, *Oxal-ac.*, *Phosph.*, *Pod.*, *Tart.*, *Verat.*, Verat-vir., *Zinc-mur.*

This reduces the list to *Canth.*, *Phosph.*, and Verat., of which only the last two have the symptom "tearing things." Both have the burning and coldness in stomach, though neither has the feeling of a cold wind. (*Coccus* has sensation of cold air blowing in stomach, but this remedy does not otherwise correspond.) Only *Veratrum* has white vomit. *Veratrum*$^{mm}$ (Fincke) once daily for a week.

May 30th reports that she took the first dose about noon of April 16th. No sickness the next day, nor any other symptom. Took no medicine second and third mornings. The third day, about three P. M., the coldness and sickness returned; she then took a dose, and in about half an hour all had gone. She then finished the medicine, taking a dose every morning, and has felt perfectly well ever since.

September 30th was safely delivered of a fine girl, the labor being short and easy. Obstetrics, like operative surgery, being a branch of the profession to which I never felt any inclination, I have not practiced as an obstetrician for very many years, except in a few special cases where my services were specially requested, and the patient made it worth my while to devote the *necessary* time to the case. So a colleague (Dr. Mary Hall) attended the accouchement. On September 6th she wrote to me: "The child has exchanged kicks for rolling itself into knobs; patient finds herself moaning, apparently from this condition." I sent one dose of Pulsat$^{cm}$ (F. C.), which removed this sensation at once.

